# Hypoglycemic Activity of Rice Resistant-Starch Metabolites: A Mechanistic Network Pharmacology and In Vitro Approach

**DOI:** 10.3390/metabo14040224

**Published:** 2024-04-15

**Authors:** Jianing Ren, Jing Dai, Yue Chen, Zhenzhen Wang, Ruyi Sha, Jianwei Mao, Yangchen Mao

**Affiliations:** 1 School of Biological and Chemical Engineering, Zhejiang University of Science and Technology, Hangzhou 310023, China; 212103817002@zust.edu.cn (J.R.); 118068@zust.edu.cn (J.D.); 212203817025@zust.edu.cn (Y.C.); 219014@zust.edu.cn (Z.W.); 101030@zust.edu.cn (J.M.); 2 School of Medicine, University of Southampton, Southampton SO17 1BJ, UK; ym8u21@soton.ac.uk

**Keywords:** rice resistant starch, hypoglycemic, network pharmacology, molecular docking, α-glucosidase, α-amylase

## Abstract

Rice (*Oryza sativa* L.) is one of the primary sources of energy and nutrients needed by the body, and rice resistant starch (RRS) has been found to have hypoglycemic effects. However, its biological activity and specific mechanisms still need to be further elucidated. In the present study, 52 RRS differential metabolites were obtained from mouse liver, rat serum, canine feces, and human urine, and 246 potential targets were identified through a literature review and database analysis. A total of 151 common targets were identified by intersecting them with the targets of type 2 diabetes mellitus (T2DM). After network pharmacology analysis, 11 core metabolites were identified, including linolenic acid, chenodeoxycholic acid, ursodeoxycholic acid, deoxycholic acid, lithocholic acid, lithocholylglycine, glycoursodeoxycholic acid, phenylalanine, norepinephrine, cholic acid, and L-glutamic acid, and 16 core targets were identified, including MAPK3, MAPK1, EGFR, ESR1, PRKCA, FYN, LCK, DLG4, ITGB1, IL6, PTPN11, RARA, NR3C1, PTPN6, PPARA, and ITGAV. The core pathways included the neuroactive ligand–receptor interaction, cancer, and arachidonic acid metabolism pathways. The molecular docking results showed that bile acids such as glycoursodeoxycholic acid, chenodeoxycholic acid, ursodeoxycholic acid, lithocholic acid, deoxycholic acid, and cholic acid exhibited strong docking effects with EGFR, ITGAV, ITGB1, MAPK3, NR3C1, α-glucosidase, and α-amylase. In vitro hypoglycemic experiments further suggested that bile acids showed significant inhibitory effects on α-glucosidase and α-amylase, with CDCA and UDCA having the most prominent inhibitory effect. In summary, this study reveals a possible hypoglycemic pathway of RRS metabolites and provides new research perspectives to further explore the therapeutic mechanism of bile acids in T2DM.

## 1. Introduction

Type II diabetes mellitus (T2DM), a complex metabolic disorder characterized by insulin resistance and impaired glucose regulation, has become a global epidemic, affecting millions of people worldwide [1]. With the escalating prevalence of unhealthy dietary habits and lifestyles, T2DM poses a significant burden on global healthcare systems, and the pathogenesis of T2DM is not fully understood in current research [2]. However, it is generally accepted that T2DM is triggered by environmental and genetic factors, including overweight, obesity, prolonged sedentary behavior, age, ethnicity, and family history [3,4]. The effective treatment of T2DM requires a comprehensive strategy that balances effectiveness with the potential to cause side effects, including nausea, vomiting, diarrhea, headaches, and even liver function abnormalities, edema, and skin reactions [5]. Relevant research suggests that dietary therapy and Chinese medicine could prevent and treat diabetes, as well as delaying the onset of complications [6,7].

Resistant starch (RS), known as anti-enzymatic starch or indigestible starch, is not digestible in the human stomach and small intestine. However, it can be fermented in the colon to produce beneficial metabolites for the health of the organism [8]. RS is generally found in foods such as cereals, vegetables, legumes, seeds, and nuts, and it is characterised by low water retention and no off-flavor, which not only improves the quality of the food, but also benefits nutrition and health [9]. In recent years, RS has attracted much attention as a novel nutritional component. Relevant studies have shown that RS has potential pharmacological effects in improving glycemic control, alleviating insulin resistance, and lowering blood lipids [10], which might work by resisting digestion, regulating the activity of enzymes related to T2DM, and modulating intestinal flora disorders [11,12,13]. Rice (*Oryza sativa* L.), as a significant source of energy, not only fulfills people’s survival needs, but also constitutes an essential part of the food culture in various countries. Rice with high-resistant-starch content has been proven to be one of the most commonly used, effective, and safe functional foods in humans, and its metabolites have positive effects on the prevention of chronic metabolic diseases [14]. Kim et al. used metabolomics to explore the effect of changes in canine fecal metabolites on obesity after the consumption of Dodamssal rice containing high levels of RS. Their results showed that Dodamssal rice remodeled the structure of the gut microbiota in obese canines, increased the contents of core metabolites such as 3-hydroxybutyric acid and 4-aminobutyric acid, and improved the symptoms of obesity and hyperglycemia [15]. The results of Wan et al. indicated that the intake of high-resistance rice under a high-fat diet led to a reduction in oleic acid content in the livers of mice, which could alleviate insulin resistance and ameliorate diabetes through the linoleic acid metabolism pathway [16]. Gao et al. explored the effects of wholegrain rice on blood glucose, lipids, and related metabolites in rats, and showed that brown rice contributed to maintaining lower blood glucose levels, while the serum metabolites 2-acetylpyrazine, glutathione, phosphatidylcholine [18:2(9*Z*,12*Z*)/15:0], and phosphatidylcholine [*O*-16:0/18:2(9*Z*,12*Z*)] were negatively correlated with fasting blood glucose and mainly ameliorated hyperglycemia through the glycerophospholipid metabolism and glutathione metabolic pathways [17]. Existing evidence indicates that the metabolites of rice resistant starch (RRS) have positive regulatory effects on glycometabolism. However, the precise molecular mechanisms underlying the role of RRS metabolites in the treatment of T2DM remain unclear and require further investigation.

Network pharmacology has gained significant academic attention due to its application in emerging areas such as traditional Chinese medicine and functional food, and it offers effective tools and methods to explore the pharmacodynamic components and mechanisms of action and their potential interactions [18]. Network pharmacology provides a theoretical foundation for drug discovery and development by integrating knowledge from various disciplines, such as biology, chemistry, and informatics. It constructs molecular interaction networks to reveal the intricate relationships between drugs and biomolecules, including targets, proteins, and genes, and has emerged as a potent tool for investigating mechanisms of drug action and disease treatment [19]. Currently, the focus of network pharmacology primarily lies in the chemical components contained within the target product. There is limited attention paid to the impact of metabolites from target products on their targets. To our knowledge, studies on the hypoglycemic properties of RS and its metabolites with a focus on network pharmacology have not been reported.

In this study, the differential metabolites of RRS were used as the research subject to predict their potential targets in T2DM therapy, while molecular docking was utilized to validate their functional efficacy, elucidating their hypoglycemic mechanisms. Furthermore, the potential mechanisms of RRS in reducing hypoglycemia were validated by inhibiting α-glucosidase and α-amylase activities in vitro.

## 2. Materials and Methods

### 2.1. Materials

α-glucosidase (50 U/mg), *p*-nitrophenyl-α-glucopyranoside (*p*-NPG), glycoursodeoxycholic acid, chenodeoxycholic acid, ursodeoxycholic acid, lithocholic acid, deoxycholic acid, and cholic acid were purchased from Shanghai Macklin Biochemical Technology Co., Ltd. (Shanghai, China). α-amylase (4000 U/g) was purchased from Shanghai Yuanye Bio-Technology Co., Ltd. (Shanghai, China). Acarbose, soluble starch, and dimethyl sulfoxide (DMSO) were purchased from Shanghai Aladdin Biochemical Technology Co., Ltd. (Shanghai, China). The other chemicals and reagents were of analytical grade and were purchased from Sinopharm Chemical Reagent Co., Ltd. (Shanghai, China).

### 2.2. Screening of Differential Metabolites and Predicting Targets of RRS

Differential metabolites of RRS in the organism were selected as active ingredients in this study. In Chinese and English literature databases, such as CNKI (https://www.cnki.net (accessed on 26 May 2023)), Web of Science (https://www.webofscience.com (accessed on 26 May 2023)), and PubMed (https://pubmed.ncbi.nlm.nih.gov (accessed on 26 May 2023)), the keywords “rice starch”, “rice resistant starch”, and “T2DM” were used to screen differential metabolites from different sources to ensure that the results pertained to the active substances of RRS. The 2D structures of the metabolites were obtained from the PubChem database (https://pubchem.ncbi.nlm.nih.gov (accessed on 28 May 2023)). Further screening was conducted using SwissADME (http://www.swissadme.ch (accessed on 28 May 2023)), which required at least two of the five principles of drug-like property principles (Lipinski, Ghose, Veber, Egan, and Muegge) to be “yes” and the degree of gastrointestinal absorption to be “high”. The 2D structures of the target metabolites were imported into the Swiss Target Prediction database (http://www.swisstargetprediction.ch (accessed on 28 May 2023)) and screened by probability > 0.1.

### 2.3. Screening of Common Targets of Metabolites and T2DM

Potential targets related to T2DM were searched in the GeneCards (https://www.genecards.org (accessed on 17 May 2023)) and OMIM databases (https://omim.org (accessed on 17 May 2023)) with the keywords “T2DM” and “type 2 diabetes”. Only targets with a “Relevance score” greater than 15 in GeneCards were considered. Subsequently, the collected targets were pooled and intersected, and duplicates were removed to obtain the final screening targets. Venny 2.1 (https://bioinfogp.cnb.csic.es/tools/venny (accessed on 25 May 2023)) was used to create a Venn diagram of intersecting targets between metabolites of RRS and T2DM. Additionally, Cytoscape 3.10.1 was used to construct a network of drugs, active ingredients, targets, and diseases. The key metabolites were selected based on their “Degree” values, which were greater than twice the median.

### 2.4. Construction of Protein–Protein Interaction (PPI) Network and Screening of Key Targets

To construct a PPI network, the intersecting targets of metabolites and diseases were imported into the “Multiple Proteins” module of the STRING database (https://cn.string-db.org (accessed on 30 May 2023)), and the species “Homo sapiens” was selected. The setting options were set as follows: we chose “full STRING network” for network type, “evidence” for the meanings of network edges, “highest confidence (0.900)” for the minimum required interaction score, and “enable 3D bubble design” and “hide disconnected nodes in the work” for the network display options. Subsequently, the node data were imported into Cytoscape 3.10.1 for visualization and network topology analysis, and the core targets were selected based on their “Degree” values, which were greater than twice the median.

### 2.5. Gene Ontology (GO) and Kyoto Encyclopedia of Genes and Genomes (KEGG) Pathway Analysis

The DAVID database (https://david.ncifcrf.gov (accessed on 30 May 2023)) was used for GO enrichment and KEGG pathway analysis. Intersecting targets of metabolites and diseases were imported into the DAVID database to explore potential pathways by which RRS may interfere with T2DM. Biological processes (BP), cellular components (CC), and molecular functions (MF) were selected for GO functional enrichment analyses, as well as KEGG pathway enrichment. Further screening was performed at *p* < 0.05.

### 2.6. Construction of Component–Target–Pathway–Disease

The core metabolites of RRS, key common targets, important pathways, and T2DM were imported into Cytoscape 3.10.1 to establish a network. Topology analysis was then performed to explore the intervention pathways of RRS in T2DM.

### 2.7. Molecular Docking of Core Metabolites of RRS and Key Targets

Molecular docking was used to simulate and verify the mechanism of action of RRS in improving T2DM. The core metabolites of RRS were used as ligands, and the core targets were used as receptors. The 2D structures of the key metabolites of RRS were downloaded from the PubChem database, and then imported into Chem3D software to generate the 3D structures with minimum free-energy treatment. Additionally, the protein structures of the targets were obtained from the PDB database (http://www.rcsb.org (accessed on 29 March 2024)). Water molecules and residues were removed using pymol software, and the hydrogenation process was performed using AutoDock software to determine the active pockets of the receptor [20]. Before formal docking, the original ligands were extracted from the target proteins and re-docked using the pre-determined method to evaluate the reliability and accuracy of AutoDock Vina in the structural docking of each protein. The root-mean-square deviations (RMSDs) between the docked ligands and the original ligands were then calculated, and the RMSD was less than 2 Å indicating high accuracy of the docking method. Following the evaluation, AutoDock Vina was used for molecular docking, and the docking results were presented through pymol software.

### 2.8. Molecular Docking of Core Metabolites of RRS, α-Glucosidase, and α-Amylase

Hypoglycemic experiments were conducted in vitro to validate the hypoglycemic activity of the key RRS metabolites which were strongly bound to core targets in the molecular docking results. Before the experiments, molecular docking was used to preliminarily determine the binding ability of key RRS metabolites to α-glucosidase and α-amylase. The metabolites derived from 2.7. with the highest ranking were used as ligands for docking, and the receptor protein structures of α-glucosidase and α-amylase were downloaded from the PDB database (http://www.rcsb.org (accessed on 29 March 2024)). The same methods were used to preprocess ligands and receptors. AutoDock Vina was used for molecular docking, and the docking results were presented through pymol software.

### 2.9. Assay of Inhibition of α-Glucosidase by Core Metabolites

The α-glucosidase inhibition of core metabolites was determined according to the method described by Tian et al. [21]. In a 96-well plate, 20 μL of sample solutions with varying concentrations (1 mg/mL, 2 mg/mL, 5 mg/mL, 10 mg/mL, 20 mg/mL) prepared in DMSO, 20 μL of 0.5 U/mL α-glucosidase, and 100 μL of 0.1 mol/L phosphate buffer (pH 6.8) were mixed and incubated for 10 min at 37 °C. Then, 20 μL of 2.5 mmoL/L *p*-Nitrophenyl-α-D-glucopyranoside (*p*NPG) was added and incubated for 15 min at 37 °C. Finally, the reactions were terminated by adding 40 μL of 0.2 moL/L NaOH solution, and the absorbance values were measured at 405 nm to evaluate the enzyme activity and calculate the enzyme inhibition. Additionally, α-glucosidase was replaced with an equal volume of phosphate buffer (0.1 mol/L, pH 6.8) to exclude its effect on the reaction. An equal volume of ultrapure water was used as a blank control for the samples, and acarbose was used as a positive control drug. Three parallels were set up in each group, and α-glucosidase inhibition was calculated with Equation (1):

(1) $$Inhibiton\;rate\;(\%)=\frac{A_{0}-A_{2}+A_{1}}{A_{0}}\;\times \;100\%$$

where A_0_ represents the acarbose of blank control, A_1_ represents the acarbose of the groups without α-glucosidase, and A_2_ represents the acarbose of the groups of experimental samples.

### 2.10. Assay of Inhibition of α-Amylase by Core Metabolites

The α-amylase inhibition of core metabolites was determined according to the method described by Wang et al. [22]. A total of 500 μL of each of sample solutions with varying concentrations (1 mg/mL, 2 mg/mL, 5 mg/mL, 10 mg/mL, 20 mg/mL) prepared in DMSO and 500 μL of 0.5 U/mL α-amylase were mixed and incubated for 10 min at 37 °C. Then, 500 μL of 1% starch solution was added and reacted for 10 min at 37 °C. Subsequently, the reaction was terminated by adding 1 mL of dinitrosalicylic acid and heated in boiling water for 5 min. After cooling to room temperature, ultrapure water was added until the reaction system reached 10 mL, and the absorbance values were measured at 540 nm to evaluate the enzyme activity and calculate the enzyme inhibition. Additionally, α-amylase was replaced with an equal volume of phosphate buffer (0.1 mol/L, pH 6.8) to exclude its effect on the reaction. An equal volume of ultrapure water was used as a blank control for the samples, and acarbose was used as a positive control drug. Three parallels were set up in each group, and the α-amylase inhibition was calculated using Equation (2):

(2) $$Inhibiton\;rate\;(\%)=\frac{A_{0}\prime -A_{2}\prime +{\;A}_{1}\prime }{A_{0}\prime }\;\times \;100\%\;$$

where A_0_′ represents the acarbose of the blank control, A_1_′ represents the acarbose of the groups without α-amylase, and A_2_′ represents the acarbose of the groups of experimental samples.

### 2.11. Statistical Analysis

The experimental data were statistically analyzed by Excel 2019 and IBM SPSS Statistics 26. The intergroup statistical differences were analyzed by using Ducan’s test, and GraphPad Prism 8 was used for plotting the results. All data are expressed as means ± SD, and *p* < 0.05 was considered to be statistically significant.

## 3. Results and Discussion

### 3.1. Metabolites and Potential Targets of RRS

A total of 93 metabolites were obtained from the literature, including 19 serum metabolites, 52 fecal metabolites, 12 hepatic metabolites, and 10 urinary metabolites, and the species sources were mice, rats, canines, and humans [15,16,17,23]. As shown in Table S1, there were 3 saccharides, 3 alcohols, 24 organic acids, 24 amino acids, 17 fatty acids, 14 alkaloids, and 8 other compounds in the selected differential metabolites. Galactose-6-phosphate is an intermediate product of galactose metabolism and participates in the glycolytic pathway in galactose metabolism. Additionally, it has been demonstrated that the oral administration of small amounts of fructose can reduce postprandial blood glucose, insulin, and C-peptide fluctuations, thereby improving diabetes [24]. The impaired metabolism that occurred in diabetics, such as increased gluconeogenesis and slowed glycolysis, could influence inositol metabolism [25]. Additionally, lipid metabolism had also been impaired in diabetic patients, which could lead to dyslipidemia, affecting glycerol metabolism [26]. Short-chain fatty acids (SCFAs) such as acetic acid, propionic acid, and butyric acid could regulate appetite and increase insulin secretion by protecting pancreatic islet tissue from damage and activating the GLP-1 signaling pathway to alleviate T2DM [27,28]. Additionally, SCFAs were also found to regulate intestinal flora disorders, which had a positive effect on improving diabetes [29]. Bile acids have been proven to activate receptors (such as FXR and TGR5) to improve glucose tolerance, insulin sensitivity, and energy metabolism [30]. Similarly, drugs like bile acid sequestrants were found to modulate glucose metabolism by improving insulin resistance [31]. In addition, metabolites such as pyruvic acid and succinic acid are involved in glycolytic metabolism and tricarboxylic acid cycle, which are closely associated with energy production and the development of metabolic diseases such as diabetes [32]. Diabetic retinopathy (DR) is a common microvascular complication of diabetes. Metabolites such as L-glutamine, L-lactate, pyruvic acid, acetic acid, L-glutamate, D-glucose, L-alanine, L-threonine, citrulline, L-lysine, and succinic acid have been identified as recurrent potential biomarkers of DR, suggesting that DR could be mitigated through specific amino acid and energy metabolic pathways [33]. Palmitoleic acid and oleic acid appeared multiple times as differentiated metabolites in an organism after interventions with high-resistance rice. There is evidence that palmitoleic acid is able to alleviate insulin resistance by activating macrophages and influencing glucose-metabolizing enzyme activities to enhance glycemic metabolism, while oleic acid primarily contributes to lipid metabolism regulation and reduces the risk of cardiovascular complications [15,34]. Alkaloids, a class of nitrogen-containing organic compounds, are widely distributed in plants. Jiao et al. detected an increase in lysophosphatidylcholine (LysoPC) and phosphatidylcholine levels in T2DM mice, which potentially indicated the progression of inflammation and reduced insulin secretion in the organism [35]. Additionally, reduced levels of unsaturated fatty acids such as eicosapentaenoic acid, arachidonic acid, and 20-hydroxyeicosatetraenoic acid were observed in this study. These results suggest that these metabolites might play significant roles in the development of diabetes. Obviously, this evidence confirms the rationality of selected differential metabolites of RRS, and they could be used in the subsequent network pharmacology studies.

Subsequently, 93 metabolites were input into the SwissADME database for further refinement based on the five principles of drug-likeness (Lipinski, Ghose, Veber, Egan, and Muegge) and gastrointestinal absorption capacity. As a result, 52 metabolites were finally selected, and detailed information on them is presented in Table 1. Then, 2D structures of the 52 target metabolites of RRS were imported into the Swiss Target Prediction database. A probability > 0.1 was used as the criterion for filtering the target compounds, and 246 potential targets were identified.

### 3.2. Screening of Intersection Targets between T2DM and Metabolites of RRS

**Figure 1:**
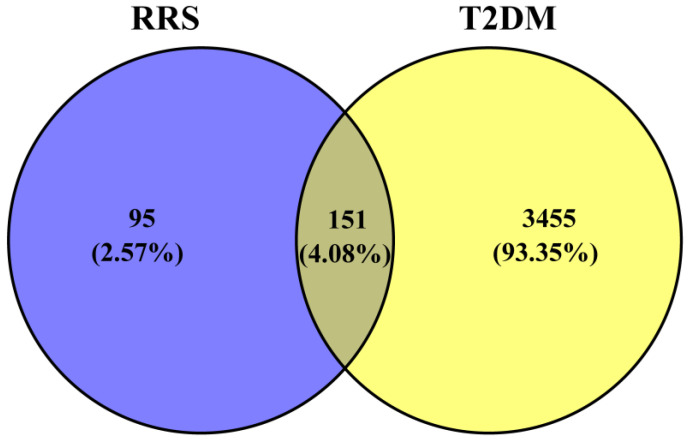
Venn diagram of intersecting targets between T2DM and metabolites of RRS.

The key words “T2DM” and “type 2 diabetes” were used to search the GeneCards and OMIM databases to identify the targets related to T2DM. As a result, 7496 potential targets were obtained. Subsequently, the intersecting targets from the GeneCards database with a “relevance score” greater than 15 and those from the OMIM database, after removing duplicates, resulted in the identification of 3606 potential therapeutic targets for T2DM. Venny 2.1 was then used to create a Venn diagram of intersecting targets between metabolites of RRS and T2DM, which is shown in Figure 1.

**Figure 2:**
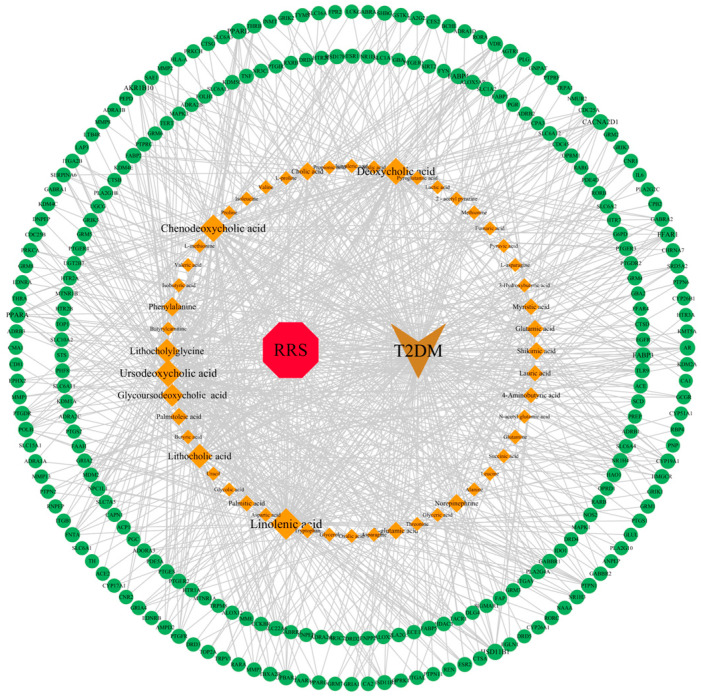
Network of RRS, metabolites, targets, and T2DM.

RRS, metabolites of RRS, common targets, and T2DM were imported into Cytoscape 3.10.1 to produce a “drug-active ingredient-target-disease” network. The result is shown in Figure 2, where the red part represents RRS; the orange part represents the metabolites of RRS, and the graphic and font sizes are positively correlated with the “Degree” values; the green part represents the common targets of RRS and T2DM; and the brown part represents T2DM. The network diagram was subjected to topological analysis, and the core metabolites of RRS were screened on the basis of the “Degree” values which were greater than twice the median. As a result, 11 metabolites were selected as the key active components, namely linolenic acid, chenodeoxycholic acid (CDCA), ursodeoxycholic acid (UDCA), deoxycholic acid (DCA), lithocholic acid (LCA), lithocholylglycine (GLCA), glycoursodeoxycholic acid (GUDCA), phenylalanine, norepinephrine, cholic acid (CA), and L-glutamic acid.

### 3.3. Construction and Analysis of PPI Protein Interaction Network

**Figure 3:**
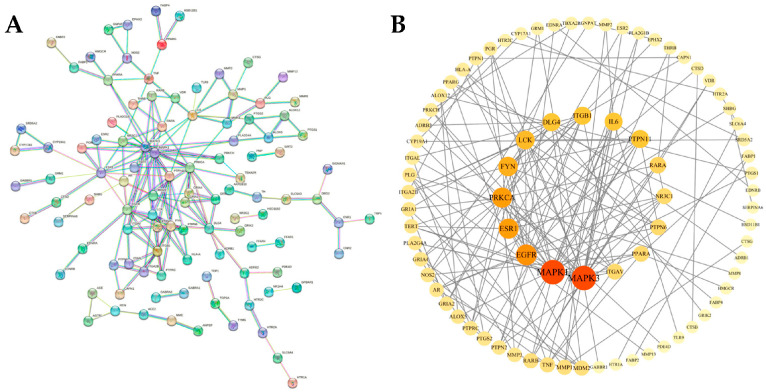
Original PPI network diagram (**A**) and topological analysis diagram (**B**) of potential targets for the metabolites of RRS.

A total of 151 common targets of metabolites of RRS and T2DM were imported into the STRING database for PPI network construction (Figure 3A), in which, the species selected was “Homo sapiens”, the isolated nodes were hidden, and the high-confidence score was set to 0.9. Then, the result of the PPI network was input into Cytoscape 3.10.1 for network topology analysis.

The result of the PPI protein interaction topology analysis is shown in Figure 3B. The “Degree” values reflect the strength of the role between each target, with darker colors and larger fonts indicating higher strength. The result indicated that there were 151 nodes and 180 edges in the network, with an average node “Degree” value of 2.38. Based on the principle of “Degree” values which were greater than twice the median, the following 16 key targets were further screened (Table 2): MAPK3, MAPK1, EGFR, ESR1, PRKCA, FYN, LCK, DLG4, ITGB1, IL6, PTPN11, RARA, NR3C1, PTPN6, PPARA, and ITGAV.

### 3.4. GO Functional Annotation and KEGG Pathway Enrichment Analysis

**Figure 4:**
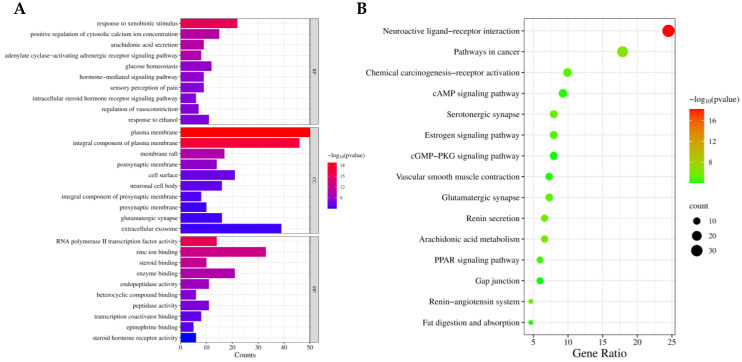
Enrichment results of main GO function annotations (**A**) and KEGG pathways (**B**).

The common targets of the metabolites of RRS and T2DM were entered into the DAVID database for GO functional annotation and KEGG pathway enrichment analysis. After screening, a total of 660 GO enrichment results were obtained, including 454 biological processes, 82 cellular compositions, and 124 molecular functions. Then, the three types of GO enrichment results were sequentially sorted from small to large according to the *p* value, and the top 10 entries in each group were selected to draw a histogram, which is shown in Figure 4A. The horizontal axis of the diagram indicates the number of enriched genes (counts), the vertical axis is −log^10^ (*p* value), and the colors from red to blue represent *p* values from small to large. The result showed that the response to xenobiotic stimulus, the positive regulation of cytosolic calcium ion concentration, and arachidonic acid secretion were mainly involved in biological processes; the plasma membrane, integral component of the plasma membrane, and membrane raft were the main cellular compositions; and RNA polymerase II transcription factor activity, ligand-activated sequence-specific DNA binding, zinc ion binding, and steroid binding were the main molecular functions.

A total of 103 KEGG pathway enrichment results were obtained, and the top five metabolic pathways were neuroactive ligand-receptor interaction, pathways in cancer, arachidonic acid metabolism, the renin–angiotensin system, and the renin secretion pathway, indicating that the metabolic components of RRS in organisms could ameliorate T2DM by regulating the related genes of these pathways. Additionally, the first 15 metabolic pathways were selected to be represented in KEGG bubble diagrams, and the specific results are shown in Figure 4B, where the horizontal axis represented the gene ratio, which indicated the proportion of genes related to the metabolic pathway to the total number of genes, and the vertical axis was −log10 (*p* value); the colors from red to green represent *p* values from small to large. The results are detailed in Table 3.

### 3.5. Construction of RRS-Metabolite-Target-Pathway-T2DM Network

**Figure 5:**
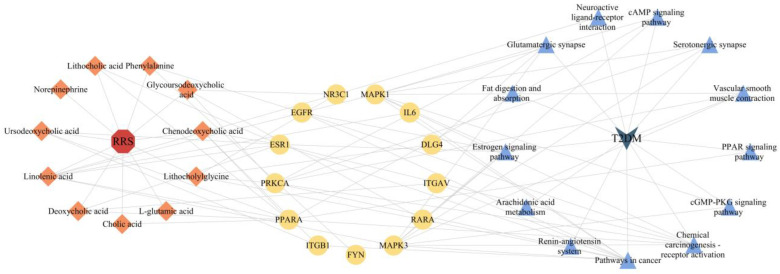
Network among RRS, metabolites, targets, pathways, and T2DM.

In total, 11 core RRS metabolites, 16 core common targets, and 15 main metabolic pathways were imported into Cytoscape 3.10.1 to construct the “RRS-metabolite-target-pathway-T2DM” network, which is shown in Figure 5. Topology analysis showed that there were 39 nodes and 88 edges in the network. The red part in the figure represents RRS, the orange part represents the core metabolites of RRS, the yellow part represents the core targets of “drug-disease”, the blue part represents the metabolic pathways of RRS in regulating T2DM, and the green part represents T2DM.

### 3.6. Verification of Molecule Docking between Core Metabolites of RRS and Targets

**Figure 6:**
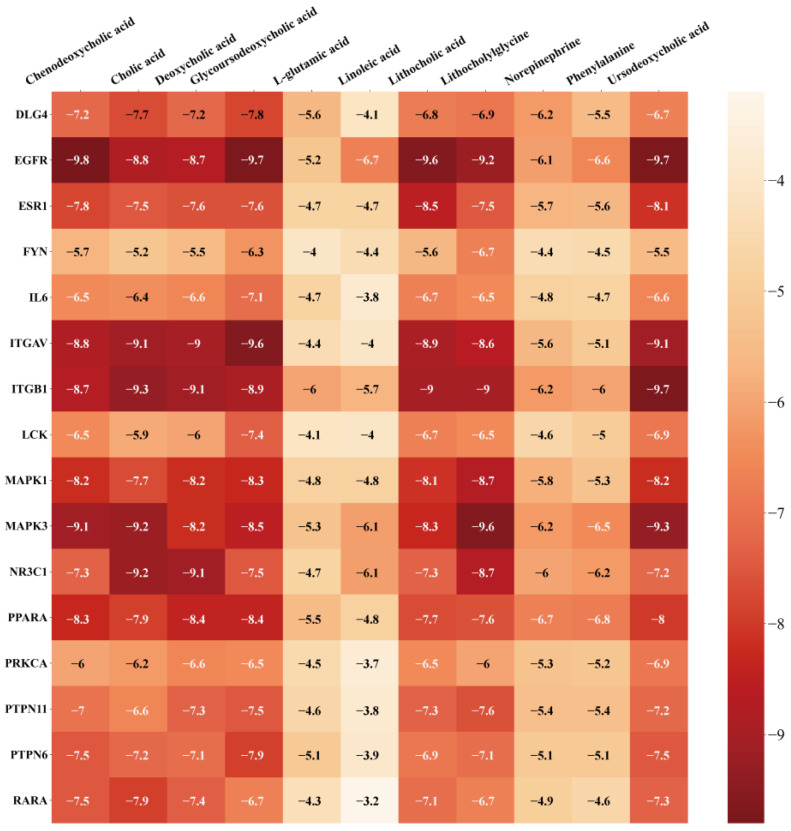
Heatmap of molecular docking results.

**Figure 7:**
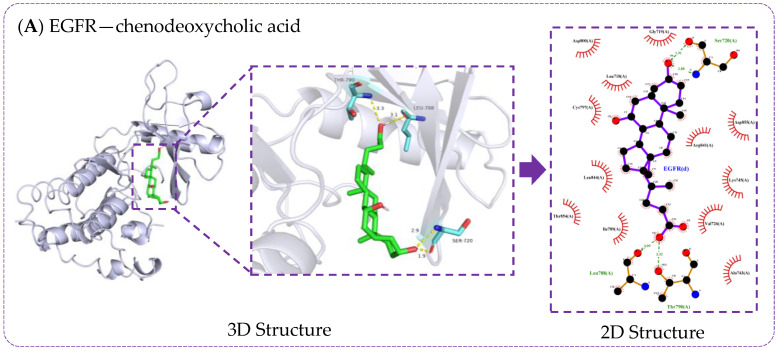

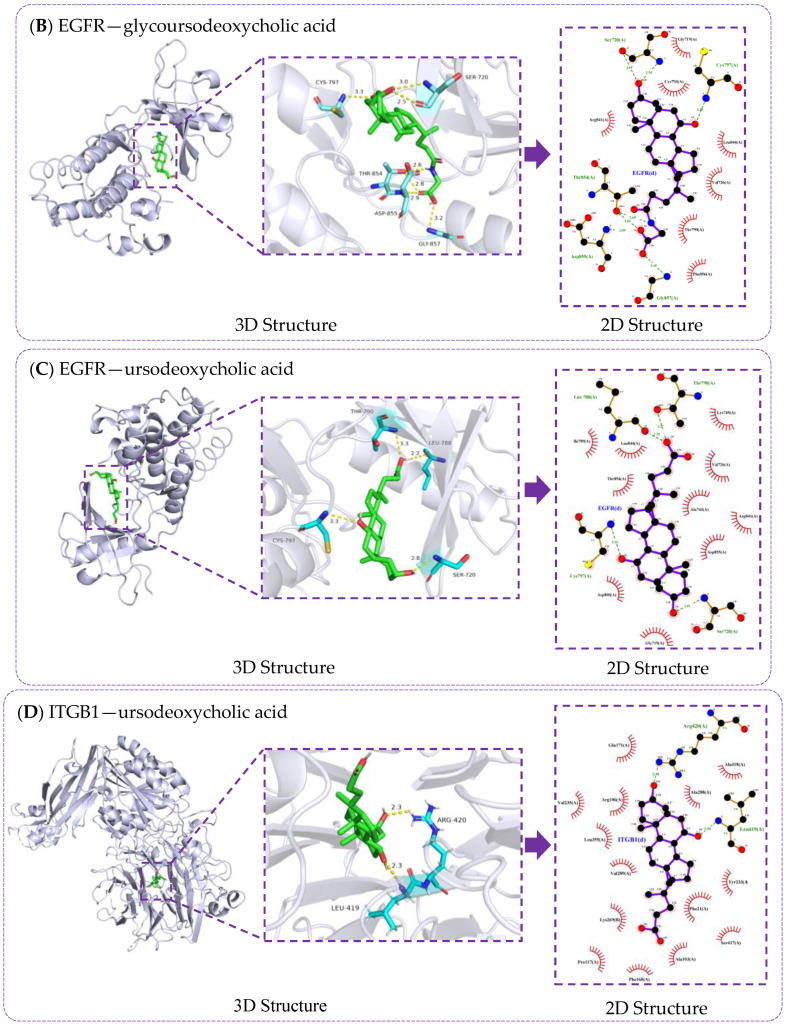

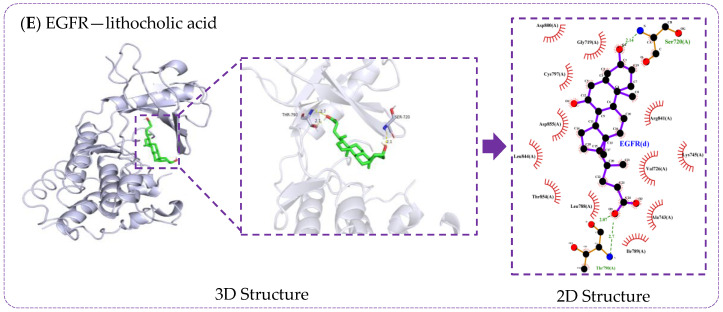
Optimization models of molecular docking.

Molecular docking was verified using AutoDock Vina software to simulate the process of the regulation of T2DM by RRS metabolites through key targets. Specific information and sources on the proteins obtained in the PDB database are shown in Table S2 [36,37,38,39,40,41,42,43,44,45,46,47,48,49,50,51]. The results of molecular docking were evaluated based on the binding free energies, and lower binding free energies represent a better binding ability of the ligand and receptor. Generally, a binding energy below −4.25 kcal/moL indicates that there is binding capacity between the ligand and the receptor, a binding energy below −5 kcal/moL indicates good binding capacity between the ligand and the receptor, and a binding energy below −7 kcal/moL indicates strong binding capacity between the ligand and the receptor [52]. The molecular docking results of the 11 RRS metabolites with 16 core targets are shown in Figure 6. All metabolites and targets were freely bound to each other, and most of the binding energies were below −4.25 kcal/moL. Among the RRS metabolites, the potential biological activities of CDCA, CA, DCA, GUDCA, LCA, and UDCA were higher. Meanwhile, EGFR, ITGAV, ITGB1, MAPK3, and NR3C1 had the best binding abilities to core metabolites. Furthermore, visualization using PyMOL software better illustrated the docking information of the top five metabolites and core targets in terms of docking energy (Figure 7). The results provide detailed insights into ligand–receptor docking, with the yellow lines in the 3D structure representing hydrogen bonds, while the 2D structure primarily depicts the hydrogen bonds and hydrophobic interactions.

The results (Figure 7 and Figure S1) show that the interaction between bile acids and EGFR protein was mainly achieved through linking with amino acid residues such as Thr790, Leu788, Ser720, Cys797, Thr854, Asp855, and Gly857. Cys797 and Thr790 have been identified as binding sites for T790M mutation inhibitors in the EGFR T790M/L858R protein (PDB ID 5HG7) [47]. Additionally, Yun et al. demonstrated that gefitinib and the AEE788 inhibitor acted on the amino acid residues Thr790, Leu788, Thr854, and Asp855 to inhibit EGFR kinase mutations [53]. The interaction between bile acids and ITGB1 protein was mainly achieved by linking to Arg420, Leu419, Glu171, and Arg106. The Arg420 of ITGB1 was able to form a salt-bridge interaction with the Asp residue in the Arg-Gly-Asp sequence, facilitating their binding [54]. Similarly, ITGAV is a member of the integrin family that facilitates cell adhesion to the extracellular matrix by recognizing ligands containing the RGD sequence, and Arg and Asp residues might play an important role in this process. Bile acids’ sites of hydrogen bonding to the MAPK3 protein include Glu126, Asp123, Glu50, Lys131, Ala52, Met125, Ile48, and Asn171. The same active sites appeared upon the binding of kaempferol 3-rutinoside-4′-glucoside to the MAPK3 protein, where Ala52, Lys131, Ile48, Asn171, and Glu50 were bound in the hydrogen-bonding mode, and van der Waals forces were present between Met125, Glu126, and Asp123 and the ligand [55]. Cholic acid was bound to NR3C1 via the amino acid residue Arg611, which had the same active site as hydrocortisone, which was bound to the EGFR protein (PDB ID 4P6X).

Core bile acids were able to bind strongly to the five core targets, and most of the binding sites were consistent with the known binding sites, indicating that the molecular docking results were reliable. Furthermore, the RMSDs of the original ligands for the five key targets after docking were less than 2 Å (Table S3), suggesting that the docking methods and parameters were reasonably designed, and the docking results were highly reliable.

It is noteworthy that bile acids constituted a significant portion of the bioactive fraction within the metabolites of RRS screened by molecular docking. Among them, CA, DCA, and CDCA belonged to primary bile acids, while LCA, GLCA, GUDCA, and UDCA were secondary bile acids. Studies have indicated an association between T2DM and bile acid metabolism, and bile acids have been found to regulate T2DM by reshaping the gut microbiota, enhancing bidirectional communication in the gut–liver axis, and reducing the expression of inflammatory factors [56].

Bile acids are synthesized by the liver, and their physiological functions include the absorption of nutrients and fat-soluble vitamins in the intestinal tract, facilitating the metabolism of lipids and toxic substances, and regulating glucose metabolism through various mechanisms [57]. Bile acids can enhance insulin sensitivity through glucose and lipid metabolism mediated by receptors. FXR is the main nuclear receptor in the physiological role of bile acids, and its role in maintaining the homeostasis of glucose and lipid metabolism in organisms had been well established [58]. The results of Han et al. demonstrated that FXR agonists improved impaired fasting glucose tolerance and alleviated symptoms associated with T2DM in FXR-knockout mice [59]. GUDCA is a novel endogenous FXR antagonist. In a study that examined the influence of metformin on T2DM via intestinal flora, the oral administration of metformin attenuated related T2DM symptoms, accompanied by a decrease in the abundance of *Bacteroidetes*, a reduction in bile salt hydrolase activity, and an increase in GUDCA levels. Therefore, the synthetic metabolism–GUDCA–gut FXR axis might represent an effective way to ameliorate the dysfunction of glucose metabolism [60]. TGR5 is a well-studied membrane-bound G-protein-coupled receptor, and its pathways for improving glucose and lipid metabolism mainly includes promoting hepatic glycogen synthesis and insulin sensitivity, increasing insulin secretion, enhancing energy expenditure, and increasing satiety [61]. Numerous studies have indicated that the TGR5 receptor activated by bile acid participates in the regulation of glycolipid metabolism processes in various tissues [62,63,64].

Moreover, bile acids have the potential to improve T2DM by reducing the concentration of cellular inflammatory factors. LCA, a product of CDCA metabolism, falls into the category of secondary bile acids. Studies have shown that LCA inhibits the production of TNF-α and IL-1β by macrophages through the activation of FXR and TGR5 receptors, thus alleviating the inflammatory response [65,66]. Additionally, DCA similarly inhibits TNF-α production by activating FXR receptors. Additionally, UDCA synthesized by intestinal flora such as *Ruminococcus* was found to play a role in regulating the immune system, which was achieved by reducing cytokine secretion by lymphocytes and inhibiting eosinophil activation and granule release [67]. In our molecular docking results, the inflammatory protein IL-6 exhibited low binding affinity with most bile acids, with the strongest affinity observed with GUDCA (−7.1 kcal/mol), suggesting that RRS might alleviate T2DM symptoms by inhibiting the expression of IL-6 via GUDCA metabolism in the body.

EGFR is a tyrosine kinase receptor that activates intracellular signaling pathways by binding to ligands such as epidermal growth factor (EGF) and affected processes such as cell proliferation, differentiation, migration, and apoptosis [68]. The interaction between enhancers and their target genes plays a significant role in gene regulation and disease pathogenesis. The study of Yang et al. aimed to determine the genetic relationship between enhancers and their target genes associated with T2DM, and resulted in the identification of a pair of SNPs, rs4947941 and rs7785013, which were significantly associated with T2DM (*p* = 4.84 × 10^−10^) [69]. *EGFR* expression was significantly upregulated in T2DM patients, consistent with the effects of rs4947941 and rs7785013 on T2DM and *EGFR* expression, suggesting that *EGFR* might be a novel T2DM susceptibility gene. Our molecular docking results showed that the binding energy of CDCA, GUDCA, and UDCA to EGFR was lower than −9.5 kcal/mol, indicating that EGFR protein might also play important role in regulating T2DM.

ITGAV and ITGB1 are two important members of the integrin family which play key roles in cell–matrix interactions and are involved in a variety of physiological and pathological processes. In T2DM, hyperglycemia is one of the main features, and prolonged hyperglycemia could cause direct damage to the kidneys, leading to diabetic nephropathy [70]. ITGAV promoted the epithelial cell proliferation, migration, and epithelial–mesenchymal transition of high-glucose-mediated lenses, as well as enhancing the activation of TGF-Smad signaling, which can lead to glomerulosclerosis and mesangial fibrosis, ultimately resulting in diabetic nephropathy [71]. In our study, the binding energy of GUDCA to ITGAV was −9.7 kcal/mol, suggesting that GUDCA might prevent T2DM complications by inhibiting the expression of ITGAV. ITGB1 is involved in the regulation of cell–matrix adhesion. In inflammatory states, the regulation of cell adhesion might be related to insulin function and insulin sensitivity [72]. Additionally, ITGB1 is also involved in neovascularization, a key pathological feature of diabetic microvascular complications, and an important factor in maintaining the normal physiological function of adipose tissue [73]. Therefore, ITGB1 might be a potential therapeutic target for diabetic microvascular complications and obesity. And recent bioinformatics studies have shown that *ITGB1* is a candidate gene closely related to T2DM and could play a key role in the diagnosis and treatment of T2DM [74].

NR3C1 is a glucocorticoid receptor belonging to the nuclear receptor family and regulates the production of glucocorticoids in organisms [75]. Glucocorticoids have been associated with insulin resistance, glucose homeostasis, and diabetes risk [76]. Studies have shown that elevated cortisol levels can trigger insulin resistance, impair pancreatic β-cell function, and increase the risk of developing T2DM [77,78]. Di et al. extracted berberine from the *Huanglian*, aiming to investigate its potential pharmacological mechanism for the treatment of T2DM and complications. PPI interaction network analysis revealed the potential mechanism of berberine’s anti-diabetic activity, involving core targets such as NR3C1, RXRA, and KCNQ1 [79]. In vivo experiments demonstrated increased expression of NR3C1 in the liver of T2DM mice, suggesting that inhibiting NR3C1 protein expression could aid in the treatment of T2DM. Our molecular docking results indicated that NR3C1 exhibited the best binding affinity to CA and DCA, which showed that RRS might potentially inhibit NR3C1 expression through metabolized bile acids, thereby treating symptoms associated with T2DM.

MAPK3, also known as ERK1, is a type of mitogen-activated protein kinase (MAPK), belonging to the MAPK family. MAPK3 plays an important role in cellular signaling pathways and is involved in regulating key processes such as cell growth, differentiation, and survival. A study analyzing the whole transcriptome of patients with T2DM treated with selegiline found that the continuous administration of 100 mg of selegiline over 12 weeks effectively alleviated hyperglycemic symptoms and significantly reduced the expression of the *MAPK3* gene in serum (*p* = 0.002). Meanwhile, *MAPK3* is involved in the abnormal activation of the MAPK pathway, which can induce insulin resistance and trigger T2DM. Therefore, selegiline can reduce insulin resistance by downregulating the expression of *MAPK3*, thereby achieving hypoglycemic effects [80]. Additionally, MAPK3 is often identified as a core target in network pharmacological studies on the treatment of T2DM, suggesting that MAPK3 might be a potentially important target for treating T2DM and its complications [81,82,83]. These findings are consistent with the results of the present study, indicating that RRS metabolites might exhibit promising hypoglycemic effects through interactions with the key targets.

In conclusion, our molecular docking results indicated that the core metabolites of RRS, including CDCA, CA, DCA, GUDCA, LCA, and UDCA, exhibited strong binding with the core targets EGFR, ITGAV, ITGB1, MAPK3, NR3C1, and RARA, which showed that RRS might potentially improve T2DM and associated symptoms through these metabolites and targets. Moreover, this improvement could be attributed to the ability of RRS to enhance insulin sensitivity, reduce lipid synthesis, inhibit the expression of inflammatory factors, and modulate intestinal flora disorders.

### 3.7. Molecular Docking Validation of Key RRS Metabolites with α-Glucosidase and α-Amylase

The 3D structures of α-glucosidase (PDB Entry: 3W37, PDB DOI: https://doi.org/10.2210/pdb3W37/pdb (accessed on 11 April 2024)) complexed with acarbose and human pancreatic α-amylase (PDB Entry: 1B2Y, PDB DOI: https://doi.org/10.2210/pdb1B2Y/pdb, accessed on 10 April 2024) were selected for molecular docking [84,85], and the results are shown in Table S4. The RMSDs of the original ligands for α-glucosidase and α-amylase after docking were 0.537 Å and 0.923 Å, indicating that the docking methods and parameters were reasonably designed, and the docking results were reliable.

As shown in Figure S2, CDCA, CA, DCA, GUDCA, LCA, and UDCA were predominantly linked to α-glucosidase through Tyr515, Ile106, Asn108, Arg113, Glu109, Ser497, Asp232, Arg552, Ser505, Ser430, Phe476, and Ala234. Acarbose is a carbohydrate inhibitor which can delay the breakdown of carbohydrates into glucose by α-glucosidase, maintaining blood glucose at normal levels. Tagami et al. reported that acarbose interacted with α-glucosidase through Ser497, Asp232, Arg552, Phe476, and Ala234 [85], which was similar to our results, suggesting that CDCA, CA, DCA, GUDCA, LCA, and UDCA might inhibit the activity of α-glucosidase to regulate blood glucose like acarbose. Six core metabolites of RRS were predominantly linked to α-amylase through His299, Asp300, Ser3, Thr6, Arg398, Glu233, His305, Phe406, Gly9, Arg10, Thr6, Glb7, His201, and His 101. The results of Li et al. showed that His299, Asp300, Glu233, His305, Gly9, and His201 were the binding sites between α-amylase inhibitory peptides and α-amylase [86], which indicated that six core RRS metabolites might also inhibit the activity of α-amylase through these known bonding sites to regulate blood glucose.

### 3.8. Inhibitory Effects of Bile Acids on α-Glucosidase and α-Amylase

**Figure 8:**
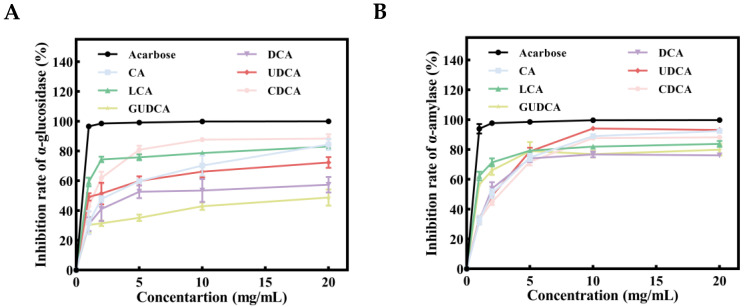

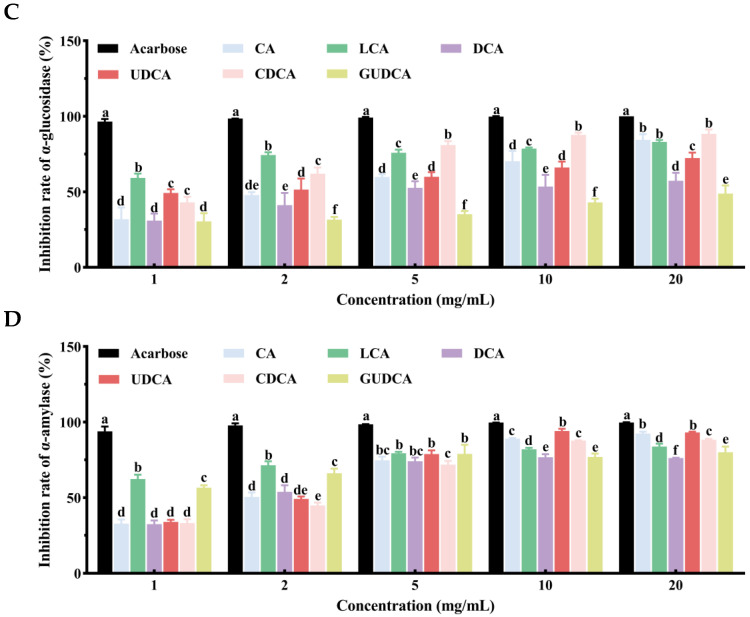
Inhibition rates of acarbose, CA, LCA, DCA, UDCA, CDCA, and GUDCA in α-glucosidase (**A**,**C**) and α-amylase (**B**,**D**). Different lowercase letters at the same time indicate significant differences between groups (*p* < 0.05).

Our molecular docking results indicated that six core metabolites of RRS exhibited potential hypoglycemic effects. To further validate the hypoglycemic effects of the core RRS metabolites, in vitro α-glucosidase and α-amylase inhibition experiments were developed. α-glucosidase served as a crucial enzyme in carbohydrate digestion and glucose breakdown. Inhibiting the expression of α-glucosidase effectively slowed down glucose uptake and regulated postprandial blood glucose levels [87]. The inhibitory effects of the core metabolites of RRS on α-glucosidase are illustrated in Figure 8A,C. The inhibition by each component shows a pattern of gradual increase followed by stabilization. At the maximum concentration (20 mg/mL), the inhibition rates of CA, LCA, DCA, UDCA, CDCA, and GUDCA were 84.43%, 83.09%, 57.34%, 72.31%, 88.37%, and 48.79%, respectively, with CA, LCA, and CDCA exhibiting the most significant inhibitory effects (*p* < 0.05). These results indicated a dose–effect relationship between the inhibitory effects of bile acids on α-glucosidase within a certain concentration range, with CDCA showing particularly pronounced inhibition, reaching a rate of 88.37% at 20 mg/mL. The overall inhibitory potency followed the order GUDCA < DCA < UDCA < LCA < CA < CDCA < Acarbose.

α-amylase is a digestive enzyme primarily active in the mouth and small intestine. The main function of it is to hydrolyze starch into glucose, and α-amylase inhibitors can slow down carbohydrate digestion in the small intestine and reduce postprandial blood glucose levels [88]. The effects of core metabolites of RRS on α-amylase inhibition are shown in Figure 8B,D. The inhibition of α-amylase by each component showed a trend of gradual increase followed by stabilization. At the maximum concentration (20 mg/mL), the inhibition rates of CA, LCA, DCA, UDCA, CDCA, and GUDCA were 92.4%, 83.75%, 76.12%, 93.15%, 88.24%, and 79.95%, respectively, with CA and UDCA exhibiting the most significant inhibition effects (*p* < 0.05). These results indicated that there was also a certain dose–effect relationship between the inhibitory effects of bile acids on α-amylase within a certain concentration range, with UDCA demonstrating particularly prominent inhibitory effects, with an inhibition rate of 93.15% at the highest concentration (20 mg/mL). The overall inhibitory capacity was classified as follows: DCA < GUDCA < LCA < CDCA < CA < UDCA < Acarbose.

Bile acids are important endogenous steroid molecules that have been proven to play a critical role in maintaining lipid levels, regulating blood glucose and energy metabolism, and preventing diabetes and obesity [89]. Activating FXR has the potential to enhance glucose metabolism. A previous reports indicated that bile acids ameliorate insulin resistance and glucose metabolism impairments in streptozotocin-induced diabetic mice by upregulating FXR expression, and the order of activation ability was CDCA > LCA = DCA > CA [90]. The results of bile acid inhibition on α-glucosidase also showed the best hypoglycemic effect of CDCA. Additionally, the intestinal bile acid–FXR signaling pathway was able to mediate the expression of FGF15/19, which bound to hepatic FGFR4/βKlotho, promoting hepatic glycogen synthesis and lowering blood glucose levels [91]. TGR5 was found to be expressed in numerous tissues and organs. Bile acids can bind to TGR5, inducing cAMP production and activating the protein kinase A pathway in various tissues and cell types, thereby regulating the organism [92]. The activation of TGR5 by bile acids in intestinal L cells induces the secretion of GLP-1 and regulates glucose metabolism by acting on pancreatic β-cells to increase insulin secretion [93]. The regulatory pathway by which metformin improves T2DM by increasing TUDCA and GUDCA levels in the intestine also involves the expression of TGR5 [60]. Additionally, bariatric surgery (MBG), such as Roux-en-Y gastric bypass (RYGB) and vertical sleeve gastrectomy (VSG), was reported to improve insulin sensitivity and diabetes. Although the underlying mechanism of diabetes remission is unclear, an increase in fasting serum bile acids, especially conjugated bile acids, might play an important role in glycemic control [94].

It is evident that CA, LCA, DCA, UDCA, CDCA, and GUDCA, as core metabolites of RRS, exhibit certain inhibitory effects on α-glucosidase and α-amylase in vitro, with CDCA and UDCA showing the most significant effects on the inhibition of α-glucosidase and α-amylase, respectively. Additionally, the results of our molecular docking validation indicated that CDCA and UDCA had the best docking affinity with EGFR, suggesting that CDCA and UDCA might be potentially important metabolites of RRS and act as hypoglycemic agents in vivo. However, current research on RS metabolites in T2DM mainly focus on SCFAs, and the specific role of bile acid metabolism and its potential in disease treatment have not been fully investigated. In addition to being involved in fat digestion and absorption, and regulating the composition of the intestinal flora, our study suggested that bile acids can control blood glucose by inhibiting the activity of digestive enzymes. Therefore, gaining a deeper understanding of the effects of RS on bile acid metabolisms is crucial, and it could also provide new strategies for the prevention and treatment of T2DM.

## 4. Conclusions

In the present study, network pharmacology was employed to predict the mechanism by which RS exerts its hypoglycemic effects through differential metabolites, which was further validated by molecular docking and hypoglycemic experiments in vitro. The results showed that CA, LCA, DCA, UDCA, CDCA, and GUDCA were the core metabolites of RRS and had great docking effects on the core targets EGFR, ITGAV, ITGB1, MAPK3, and NR3C1. RRS might have exerted hypoglycemic effects through the neuroactive ligand–receptor interaction pathway, cancer pathway, and arachidonic acid metabolic pathway. Meanwhile, core RRS metabolites, including six bile acids, were able to bind to α-glucosidase and α-amylase through known binding sites, and hypoglycemic tests in vitro showed that there was a certain dose–effect relationship between the inhibitory effect of these bile acids on α-glucosidase and α-amylase within a certain concentration range, with the prominent inhibitory effect observed for CDCA and UDCA. This study reveals the potential hypoglycemic pathway of RRS in terms of molecular mechanisms and provides a new perspective for understanding the role of bile acids in the prevention and treatment of T2DM.

## Figures and Tables

**Table 1:** The information of 52 differential metabolites screened by SwissADME.

Metabolites	Principles of Drug-Likeness					Gastrointestinal Absorption Capacity	PubChem CID
	Lipinski	Ghose	Veber	Egan	Muegge		
2-acetyl pyrazine	Yes	No	Yes	Yes	No	High	30914
3-hydroxybutyric acid	Yes	No	Yes	Yes	No	High	441
4-aminobutyric acid	Yes	No	Yes	Yes	No	High	119
Alanine	Yes	No	Yes	Yes	No	High	5950
Asparagine	Yes	No	Yes	Yes	No	High	6267
Aspartic acid	Yes	No	Yes	Yes	No	High	5960
Butyric acid	Yes	No	Yes	Yes	No	High	264
Butyrylcarnitine	Yes	No	Yes	Yes	Yes	High	213114
Chenodeoxycholic acid	Yes	Yes	Yes	Yes	Yes	High	10133
Cholic acid	Yes	Yes	Yes	Yes	Yes	High	221493
Deoxycholic acid	Yes	Yes	Yes	Yes	Yes	High	222528
Fumaric acid	Yes	No	Yes	Yes	No	High	444972
Glutamic acid	Yes	No	Yes	Yes	No	High	33032
Glutamine	Yes	No	Yes	Yes	No	High	5961
Glyceric acid	Yes	No	Yes	Yes	No	High	752
Glycerol	Yes	No	Yes	Yes	No	High	753
Glycolic acid	Yes	No	Yes	Yes	No	High	757
Glycoursodeoxycholic acid	Yes	No	Yes	Yes	Yes	High	12310288
Isobutyric acid	Yes	No	Yes	Yes	No	High	6590
Isoleucine	Yes	No	Yes	Yes	No	High	6306
Isovaleric acid	Yes	No	Yes	Yes	No	High	10430
Lactic acid	Yes	No	Yes	Yes	No	High	612
L-asparagine	Yes	No	Yes	Yes	No	High	6267
Lauric acid	Yes	Yes	Yes	Yes	Yes	High	3893
Leucine	Yes	No	Yes	Yes	No	High	6106
L-glutamic acid	Yes	No	Yes	Yes	No	High	33032
Linolenic acid	Yes	No	No	Yes	No	High	5280934
Lithocholic acid	Yes	Yes	Yes	Yes	Yes	High	9903
Lithocholylglycine	Yes	No	Yes	Yes	Yes	High	115245
L-methionine	Yes	No	Yes	Yes	No	High	6137
L-proline	Yes	No	Yes	Yes	No	High	145742
Malic acid	Yes	No	Yes	Yes	No	High	525
Methionine	Yes	No	Yes	Yes	No	High	6137
Myristic acid	Yes	Yes	No	Yes	No	High	11005
N-acetyl glutamic acid	Yes	No	Yes	Yes	No	High	70914
Norepinephrine	Yes	Yes	Yes	Yes	No	High	439260
Oxalic acid	Yes	No	Yes	Yes	No	High	971
Palmitic acid	Yes	Yes	No	Yes	No	High	985
Palmitoleic acid	Yes	Yes	No	Yes	No	High	445638
Phenylalanine	Yes	Yes	Yes	Yes	No	High	6140
Proline	Yes	No	Yes	Yes	No	High	145742
Propionic acid	Yes	No	Yes	Yes	No	High	1032
Pyroglutamic acid	Yes	No	Yes	Yes	No	High	7405
Pyruvic acid	Yes	No	Yes	Yes	No	High	1060
Shikimic acid	Yes	No	Yes	Yes	No	High	8742
Succinic acid	Yes	No	Yes	Yes	No	High	1110
Threonine	Yes	No	Yes	Yes	No	High	6288
Tryptophan	Yes	Yes	Yes	Yes	Yes	High	6305
Uracil	Yes	No	Yes	Yes	No	High	1174
Ursodeoxycholic acid	Yes	Yes	Yes	Yes	Yes	High	31401
Valeric acid	Yes	No	Yes	Yes	No	High	7991
Valine	Yes	No	Yes	Yes	No	High	6287

**Table 2:** Attribution of main targets.

Target	Betweenness Centrality	Closeness Centrality	Topological Coefficient	Degree
MAPK3	0.223813617	0.428571429	0.155555556	18
MAPK1	0.199221844	0.428571429	0.145555556	18
EGFR	0.140099305	0.384236453	0.226824458	13
ESR1	0.194078415	0.371428571	0.204545455	12
PRKCA	0.212868248	0.39	0.191056911	12
FYN	0.086891474	0.366197183	0.227272727	11
LCK	0.032375537	0.345132743	0.306666667	10
DLG4	0.19912985	0.331914894	0.189814815	9
ITGB1	0.042968916	0.320987654	0.247863248	9
IL6	0.169924934	0.352941176	0.233333333	9
PTPN11	0.044866981	0.378640777	0.287749288	9
RARA	0.040924344	0.348214286	0.3	7
NR3C1	0.064554392	0.342105263	0.369047619	6
PTPN6	0.003184922	0.310756972	0.438596491	6
PPARA	0.135433492	0.329113924	0.306666667	6
ITGAV	0.017906128	0.331914894	0.410714286	6

**Table 3:** Information of enrichment results of main KEGG pathways.

KEGG ID	Pathway	Gene Ratio (%)	*p* Value	Counts
hsa04080	Neuroactive ligand–receptor interaction	24.50331126	6.08 × 10^−19^	37
hsa05200	Pathways in cancer	17.8807947	3.93 × 10^−7^	27
hsa00590	Arachidonic acid metabolism	6.622516556	5.68 × 10^−7^	10
hsa04614	Renin–angiotensin system	4.635761589	1.42 × 10^−6^	7
hsa04924	Renin secretion	6.622516556	1.66 × 10^−6^	10
hsa04726	Serotonergic synapse	7.947019868	2.64 × 10^−6^	12
hsa05207	Chemical carcinogenesis–receptor activation	9.933774834	9.29 × 10^−6^	15
hsa04915	Estrogen signaling pathway	7.947019868	1.55 × 10^−5^	12
hsa04724	Glutamatergic synapse	7.284768212	1.66 × 10^−5^	11
hsa03320	PPAR signaling pathway	5.960264901	2.91 × 10^−5^	9

## Data Availability

The raw data supporting the conclusions of this article will be made available by the authors on request.

## Supplementary Materials

The following supporting information can be downloaded at: https://www.mdpi.com/article/10.3390/metabo14040224/s1, Figure S1: Molecular docking of core metabolites of RRS and key targets; Figure S2: Molecular docking validation of key RRS metabolites with α-glucosidase and α-amylase; Table S1: 93 differential metabolites of RRS; Table S2: Molecular docking information of target proteins; Table S3: Molecular docking information of target proteins and original ligands; Table S4: Molecular docking results of key RRS metabolites with α-glucosidase and α-amylase.
