# Supplementary material for: Hypoglycemic Activity of Rice Resistant-Starch Metabolites: A Mechanistic Network Pharmacology and In Vitro Approach
Source: Metabolites. 2024 Apr 15;14(4):224. doi: 10.3390/metabo14040224 (PMC11052319; doi:10.3390/metabo14040224)
Supplement: Supplementary file 1 [file metabolites-14-00224-s001.zip › metabolites-2923907-supplementary.pdf]

Supplementary materials

Table S1. 93 differential metabolites of RRS.

Class	Number	Differential metabolites	References
saccharide	1	Galactose	15
	2	Fructose	15
	3	Galactose-6-phosphate	16
alcohol	4	Glycerol	15
	5	Ethanolamine	15
	6	Inositol	15
Organic acid	7	3-Hydroxybutyric acid	15
	8	Malic acid	16
	9	Pyruvic acid	15
	10	Shikimic acid	15
	11	Lactic acid	15
	12	Oxalic acid	15
	13	Succinic acid	15
	14	Fumaric acid	15
	15	Glyceric acid	15
	16	Glycolic acid	15
	17	Citric acid	16
	18	Isovaleric acid	23
	19	Phosphoric acid	15
	20	Propionic acid	23
	21	Valeric acid	23
	22	Isobutyric acid	23
	23	Butyric acid	23
	24	Deoxycholic acid	23
	25	Lithocholylglycine	23
	26	Glycoursodeoxycholic acid	23
	27	Cholic acid	23
	28	Chenodeoxycholic acid	23
	29	Lithocholic acid	23
	30	Ursodeoxycholic acid	23
Amino acid	31	Serine	15
	32	Pyroglutamic acid	15
	33	Glutamine	15
	34	Alanine	15,23
	35	Asparagine	15
	36	L-asparagine	17
	37	Aspartic acid	15,23
	38	Proline	15

	39	L-proline	17
	40	Tryptophan	15
	41	Threonine	15
	42	Methionine	15
	43	L-methionine	17
	44	Leucine	15,23
	45	Isoleucine	15
	46	Valine	15,23
	47	Glycine	15,17
	48	Phenylalanine	15,23
	49	Taurine	16
	50	4-Aminobutyric acid	15
	51	Glutamic acid	15,23
	52	L-glutamic acid	17
	53	β-Alanine	15
	54	N-acetyl glutamic acid	17
Fatty acid	55	Linolenic acid	15
	56	Eicosapentaenoic acid	15
	57	Arachidonic acid	15
	58	Eicosatrienoic acid	15
	59	Palmitoleic acid	15,16,17
	60	Lauric acid	15
	61	Myristic acid	15
	62	Palmitic acid	15
	63	Clupanodonic acid	15
	64	Docosatrienoic acid	17
	65	Linoleic acid	15
	66	Oleic acid	15,16
	67	Stearic acid	15
	68	Arachidic acid	15
	69	Behenic acid	15
	70	Lignoceric acid	15
	71	Docosahexaenoic acid	15
alkaloid	72	Butyrylcarnitine	17
	73	LysoPC [16:1 (9Z)]	17
	74	LysoPC (17: 0)	17
	75	LysoPC (20:0)	17
	76	LysoPC [20:1 (11Z)]	17
	77	LysoPC [20:3 (5Z,8Z,11Z)]	17
	78	LysoPC (24:0)	17
	79	LysoPC (O-18:0)	17
	80	LysoPC (P-16:0)	17
	81	LysoPC (P-18:0)	17
	82	Phosphatidylcholine [16:1 (9Z) /16:0]	17

	83	Phosphatidylcholine [18:0/20:3 (5Z,8Z,11Z)]	17
	84	Phosphatidylcholine [18:2 (9Z,12Z) / 15:0]	17
	85	Phosphatidylcholine [O-16:0/18:2 (9Z,12Z)]	17
other	86	Urea	15
	87	Uracil	16
	88	2 - acetyl pyrazine	17
	89	Norepinephrine	17
	90	1,5-Anhydroglucitol	16
	91	Glutathione	17
	92	Ceramide (d18:0/14:0)	17
	93	Sphingomyelin (d18:1/14:0)	17

Table S2. Molecular docking information of target proteins.

Target Protein	PDB Entry	Resolution / Å	PDB DOI	References
DLG4	6QJL	1.04	https://doi.org/10.2210/pdb6QJL/pdb	[36]
EGFR	5UG9	1.33	https://doi.org/10.2210/pdb5UG9/pdb	[37]
ESR1	7UJO	1.45	https://doi.org/10.2210/pdb7UJO/pdb	[38]
FYN	6IPY	1.34	https://doi.org/10.2210/pdb7A2P/pdb	[39]
IL6	7NXZ	2.00	https://doi.org/10.2210/pdb7NXZ/pdb	[40]
ITGAV	5FFG	2.25	https://doi.org/10.2210/pdb5FFG/pdb	[41]
ITGB1	4WK0	1.78	https://doi.org/10.2210/pdb4WK0/pdb	[42]
LCK	1QPC	1.60	https://doi.org/10.2210/pdb1QPC/pdb	[43]
MAPK1	6SLG	1.33	https://doi.org/10.2210/pdb6SLG/pdb	[44]
MAPK3	4QTB	1.40	https://doi.org/10.2210/pdb4QTB/pdb	[45]
NR3C1	4P6X	1.95	https://doi.org/10.2210/pdb4P6W/pdb	[46]
PPARA	6KB1	1.25	https://doi.org/10.2210/pdb6KB1/pdb	[47]
PRKCA	2GZV	1.12	https://doi.org/10.2210/pdb2GZV/pdb	[48]
PTPN6	4GRZ	1.37	https://doi.org/10.2210/pdb4GRZ/pdb	[49]
PTPN11	3B7O	1.60	https://doi.org/10.2210/pdb3B7O/pdb	[50]
RARA	3KMR	1.80	https://doi.org/10.2210/pdb3KMR/pdb	[51]

Table S3. Molecular docking information of target proteins and original ligands.

Target Protein	PDB Entry	Original ligand	RMSD / Å
EGFR	5UG9	8AM	0.528
ITGAV	5FFG	NAG	0.751
ITGB1	4WK0	NAG	1.168
MAPK3	4QTB	38Z	0.386
NR3C1	4P6X	HCY	0.261

Table S4. Molecular docking results of key RRS metabolites with α-glucosidase and α-amylase.

Number	Ligand	Receptor	Binding energy
1	Chenodeoxycholic acid	α-glucosidase (3W37)	-7.6
2	Cholic acid	α-glucosidase (3W37)	-7.9
3	Deoxycholic acid	α-glucosidase (3W37)	-7.3
4	Glycoursodeoxycholic acid	α-glucosidase (3W37)	-8.3
5	Lithocholic acid	α-glucosidase (3W37)	-7.3
6	Ursodeoxycholic acid	α-glucosidase (3W37)	-7.9
7	Chenodeoxycholic acid	α-amylase (1B2Y)	-8.6
8	Cholic acid	α-amylase (1B2Y)	-8.6
9	Deoxycholic acid	α-amylase (1B2Y)	-8.6
10	Glycoursodeoxycholic acid	α-amylase (1B2Y)	-7.7
11	Lithocholic acid	α-amylase (1B2Y)	-8.4
12	Ursodeoxycholic acid	α-amylase (1B2Y)	-8.8

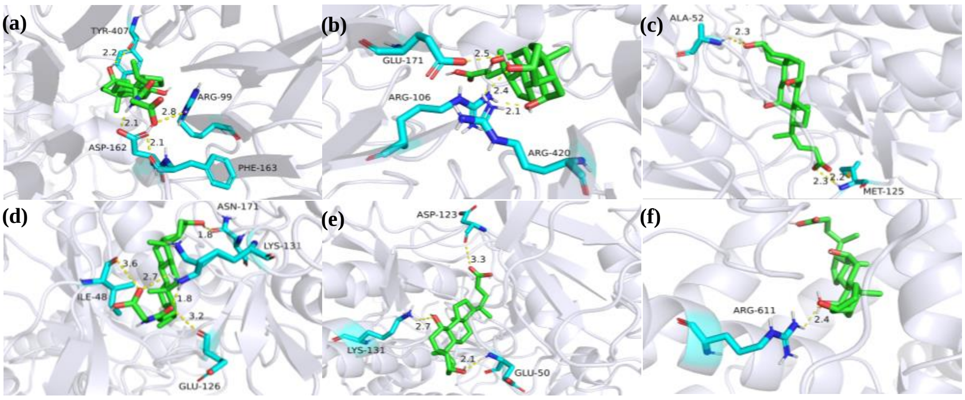

Figure S1. Molecular docking of core metabolites of RRS and key targets. (A): Glycoursodeoxycholic acid, ITGAV; (B): Cholic acid, ITGB1; (C): Cholic acid, MAPK3; (D): Lithocholylglycine, MAPK3; (E): Ursodeoxycholic acid, MAPK3; (F): Cholic acid, NR3C1.

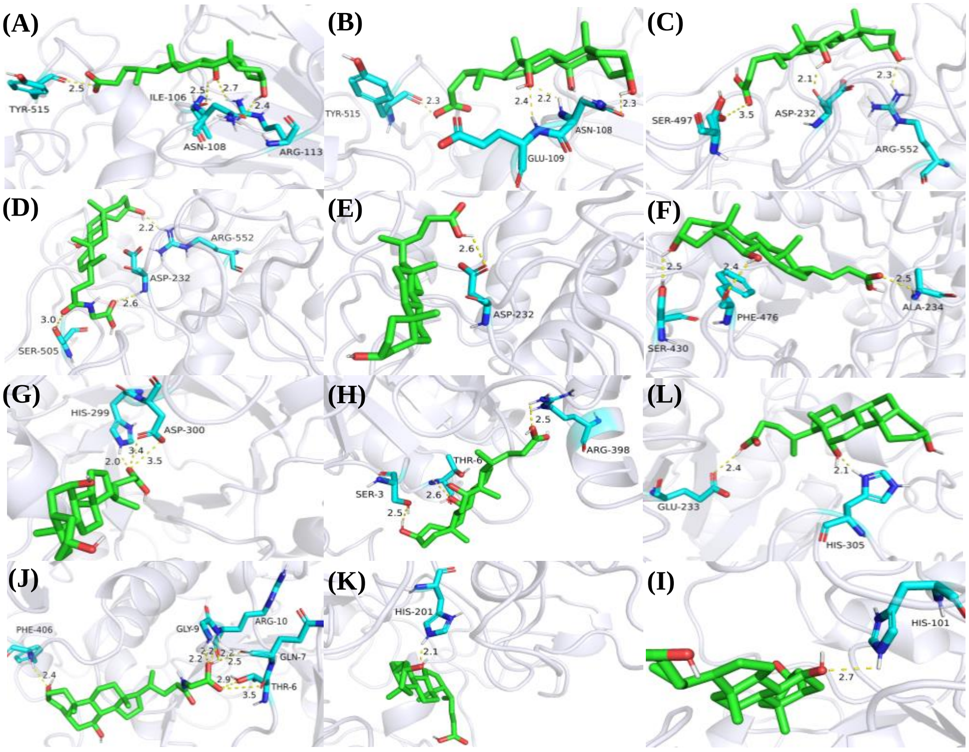

Figure S2. Molecular docking validation of key RRS metabolites with α-glucosidase and α-amylase. (A): Chenodeoxycholic acid, α-glucosidase; (B): Cholic acid, α-glucosidase; (C): Deoxycholic acid, α-glucosidase; (D): Glycoursodeoxycholic acid, α-glucosidase; (E): Lithocholic acid, α-glucosidase; (F): Ursodeoxycholic acid, α-glucosidase; (G): Chenodeoxycholic acid, α-amylase; (H): Cholic acid, α-amylase; (I): Deoxycholic acid, α-amylase; (J): Glycoursodeoxycholic acid, α-amylase; (K): Lithocholic acid, α-amylase; (L): Ursodeoxycholic acid, α-amylase.

References

15. Kim, Y.J.; Kim, J.G.; Lee, W.K.; So, K.M.; Kim, J.K. Trial data of the anti-obesity potential of a high resistant starch diet for canines using Dodamssal rice and the identification of discriminating markers in feces for metabolic profiling. *Metabolomics* **2019**, *15*, 21, doi:10.1007/s11306-019-1479-4.

16. Wan, J. Effects of Rice with Different Amounts of Resistant Starch on Mice Fed A High-fat Diet:Regulation of Lipid Metabolism and Gut Microbiota. Ph D thesis, Huazhong Agricultural University, Wuhan, 2021, doi:10.27158/d.cnki.ghznu.2020.000129.

17. Gao, P.; Zhang, Y.; Gao, C.; Xiang, X.; Zhang, X.; Wang, Z. Effects of brown rice on metabolomics related to glucose and lipid in normal rats. *Wei Sheng Yan Jiu* **2021**, *50*, 600-608, doi:10.19813/j.cnki.weishengyanjiu.2021.04.011.

23. Qian, L. Diagnosis of Non-alcoholic Fatty Liver Disease and Clinical Studies of Resistant Starch Intervention. Ph D thesis, Shanghai Jiaotong University, Shanghai, 2022, doi:10.27307/d.cnki.gsjtu.2020.000327.

36. Camara-Artigas, A.; Murciano-Calles, J.; Martínez, J.C. Conformational changes in the third PDZ domain of the neuronal postsynaptic density protein 95. *Acta Crystallogr D Struct Biol* **2019**, *75*, 381-391, doi:10.1107/s2059798319001980.

37. Planken, S.; Behenna, D.C.; Nair, S.K.; Johnson, T.O.; Nagata, A.; Almaden, C.; Bailey, S.; Ballard, T.E.; Bernier, L.; Cheng, H.; et al. Discovery of N-((3R,4R)-4-Fluoro-1-(6-((3-methoxy-1-methyl-1H-pyrazol-4-yl)amino)-9-methyl-9H-purin-2-yl)pyrrolidine-3-yl)acrylamide (PF-06747775) through Structure-Based Drug Design: A High Affinity Irreversible Inhibitor Targeting Oncogenic EGFR Mutants with Selectivity over Wild-Type EGFR. *J. Med. Chem.* **2017**, *60*, 3002-3019, doi:10.1021/acs.jmedchem.6b01894.

38. Hosfield, D.J.; Weber, S.; Li, N.S.; Sauvage, M.; Joiner, C.F.; Hancock, G.R.; Sullivan, E.A.; Ndukwe, E.; Han, R.; Cush, S.; et al. Stereospecific lasofoxifene derivatives reveal the interplay between estrogen receptor alpha stability and antagonistic activity in ESR1 mutant breast cancer cells. *Elife* **2022**, *11*, doi:10.7554/eLife.72512.

39. Aldehaiman, A.; Momin, A.A.; Restouin, A.; Wang, L.; Shi, X.; Aljedani, S.; Opi, S.; Lugari, A.; Shahul Hameed, U.F.; Ponchon, L.; et al. Synergy and allostery in ligand binding by HIV-1 Nef. *Biochem J* **2021**, *478*, 1525-1545, doi:10.1042/bcj20201002.

40. Reif, A.; Lam, K.; Weidler, S.; Lott, M.; Boos, I.; Lokau, J.; Bretscher, C.; Mönnich, M.; Perkams, L.; Schmälzlein, M.; et al. Natural Glycoforms of Human Interleukin 6 Show Atypical Plasma Clearance. *Angew. Chem. Int. Ed. Engl.* **2021**, *60*, 13380-13387, doi:10.1002/anie.202101496.

41. Dong, X.; Zhao, B.; Iacob, R.E.; Zhu, J.; Koksal, A.C.; Lu, C.; Engen, J.R.; Springer, T.A. Force interacts with macromolecular structure in activation of TGF-β. *Nature* **2017**, *542*, 55-59, doi:10.1038/nature21035.

42. Xia, W.; Springer, T.A. Metal ion and ligand binding of integrin α5β1. *Proc Natl Acad Sci U S A* **2014**, *111*, 17863-17868, doi:10.1073/pnas.1420645111.

43. Zhu, X.; Kim, J.L.; Newcomb, J.R.; Rose, P.E.; Stover, D.R.; Toledo, L.M.; Zhao, H.; Morgenstern, K.A. Structural analysis of the lymphocyte-specific kinase Lck in complex with non-selective and Src family selective kinase inhibitors. *Structure* **1999**, *7*, 651-661, doi:10.1016/s0969-2126(99)80086-0.

44. Ward, R.A.; Anderton, M.J.; Bethel, P.; Breed, J.; Cook, C.; Davies, E.J.; Dobson, A.; Dong, Z.;

Fairley, G.; Farrington, P.; et al. Discovery of a Potent and Selective Oral Inhibitor of ERK1/2 (AZD0364) That Is Efficacious in Both Monotherapy and Combination Therapy in Models of Nonsmall Cell Lung Cancer (NSCLC). *J. Med. Chem.* **2019**, *62*, 11004-11018, doi:10.1021/acs.jmedchem.9b01295.

45. Chaikuad, A.; Tacconi, E.M.; Zimmer, J.; Liang, Y.; Gray, N.S.; Tarsounas, M.; Knapp, S. A unique inhibitor binding site in ERK1/2 is associated with slow binding kinetics. *Nat. Chem. Biol.* **2014**, *10*, 853-860, doi:10.1038/nchembio.1629.

46. He, Y.; Yi, W.; Suino-Powell, K.; Zhou, X.E.; Tolbert, W.D.; Tang, X.; Yang, J.; Yang, H.; Shi, J.; Hou, L.; et al. Structures and mechanism for the design of highly potent glucocorticoids. *Cell Res.* **2014**, *24*, 713-726, doi:10.1038/cr.2014.52.

47. Kamata, S.; Oyama, T.; Saito, K.; Honda, A.; Yamamoto, Y.; Suda, K.; Ishikawa, R.; Itoh, T.; Watanabe, Y.; Shibata, T.; et al. PPARα Ligand-Binding Domain Structures with Endogenous Fatty Acids and Fibrates. *iScience* **2020**, *23*, 101727, doi:10.1016/j.isci.2020.101727.

48. Elkins, J.M.; Papagrigoriou, E.; Berridge, G.; Yang, X.; Phillips, C.; Gileadi, C.; Savitsky, P.; Doyle, D.A. Structure of PICK1 and other PDZ domains obtained with the help of self-binding C-terminal extensions. *Protein Sci.* **2007**, *16*, 683-694, doi:10.1110/ps.062657507.

49. Alicea-Velázquez, N.L.; Jakoncic, J.; Boggon, T.J. Structure-guided studies of the SHP-1/JAK1 interaction provide new insights into phosphatase catalytic domain substrate recognition. *J Struct Biol* **2013**, *181*, 243-251, doi:10.1016/j.jsb.2012.12.009.

50. Barr, A.J.; Ugochukwu, E.; Lee, W.H.; King, O.N.; Filippakopoulos, P.; Alfano, I.; Savitsky, P.; Burgess-Brown, N.A.; Müller, S.; Knapp, S. Large-scale structural analysis of the classical human protein tyrosine phosphatome. *Cell* **2009**, *136*, 352-363, doi:10.1016/j.cell.2008.11.038.

51. le Maire, A.; Teyssier, C.; Erb, C.; Grimaldi, M.; Alvarez, S.; de Lera, A.R.; Balaguer, P.; Gronemeyer, H.; Royer, C.A.; Germain, P.; et al. A unique secondary-structure switch controls constitutive gene repression by retinoic acid receptor. *Nat. Struct. Mol. Biol.* **2010**, *17*, 801-807, doi:10.1038/nsmb.1855.
